# Multiple Post-Typhoid Abscesses

**Published:** 1908-01

**Authors:** F. H. Little

**Affiliations:** Muscatine, Iowa


### CLINICAL REPORT.

### Multiple Post-Typhoid Abscesses.

By F. H. LITTLE, M. D., Muscatine, Iowa.

I RECENTLY saw, in my practice, a case of typhoid fever with a rather unusual complication, which prolonged the convalescence very materially. As I have found very little mention of similar cases in the literature, I have thought this case deserved being brought to the attention of the profession:

On May, 1, 1907, Mrs. J. G., aged forty-eight, was taken ill with typhoid fever; the typical symptoms were present and the fever ran the ordinary course, lasting for twenty-one days before reaching normal. The illness was not a severe type of the disease and was rather easily controlled.

Within a few days after temperature reached normal, she began to complain of severe pain in the calf of the left leg. The pain persisted; skin became reddened and this congested area soon developed into an abscess containing quantities of pus. This was followed by others until a total of twenty-five abscesses occurred extending from the instep to the groin. All the abscesses were freely opened and drained. The abscess cavities were irrigated twice daily with a 50% solution of hydrogen peroxide and packed with plain gauze; patient was put on a nutritious diet; careful attention was given the skin and the bowels; iodine in the form of the iodo-proteid compound, iodalbin, was given every four hours in doses of five grains, preference being given to this preparation over the pot. iodid, owing to my desire to relieve the patient of any possibility of this salt's producing the well known gastric and intestinal irritation so frequently seen after its administration. Under this treatment, the case progressed rapidly to a perfect recovery with full use of the leg, and at no time during the treatment were any gastric disturbances or skin manifestations noted from the large amount of iodalbin (5 grs. every four hours) which was given.

I believe that this case is quite remarkable, owing to the fact that twenty-five abscesses were developed upon the leg and that they yielded readily to the powerful alterative effect of the iodalbin compound with the surgical treatment mentioned. The textbooks mention nothing exactly like this case, although attention is called to the fact that during the stage of convalescence the muscles of the lower extremities may become painful, even a slight redness may appear in the skin covering the muscles, and that this is usually unilateral. Most commonly this affects the calf of the leg and pain is developed on pressure or movement. This condition is said to be due to myositis and must not be confused with phlegmasia dolens due to thrombosis. Reference is also made in our standard works to the fact that convalescence is not infrequently delayed by numerous boils and that rarely multiple abscesses occur. The etiology seems to be obscure.

